# Translational and Clinical Applications of Dental Stem Cell-Derived Exosomes

**DOI:** 10.3389/fgene.2021.750990

**Published:** 2021-10-26

**Authors:** Zizhao Mai, Huan Chen, Yu Ye, Ziyu Hu, Wenjuan Sun, Li Cui, Xinyuan Zhao

**Affiliations:** ^1^ Stomatological Hospital, Southern Medical University, Guangzhou, China; ^2^ Institute of Stomatology, Nanjing Medical University, Nanjing, China; ^3^ Key Laboratory of Oral Diseases of Jiangsu Province, Stomatological Institute, Nanjing Medical University, Nanjing, China; ^4^ Department of Pediatrics, Nanjing Jinling Stomatology Hospital, Nanjing, China; ^5^ Department of Stomatology, The Third Affiliated Hospital, Sun Yat-sen University, Guangzhou, China; ^6^ UCLA School of Dentistry, Los Angeles, CA, United States

**Keywords:** dental stem cells, exosomes, regenerative medicine, tissue repair, clinical application

## Abstract

Mesenchymal stem cells (MSCs) are promising seed cells in tissue repair and regeneration due to their featured properties of self-renewal and multipotency. However, a growing body of evidence has demonstrated that MSCs exert biological functions mainly through secreting exosomes. Exosomes, which contain RNA, proteins, lipids, and metabolites, are new players in regulating many fundamental processes and play important roles in regenerative medicine. Exosomes not only mimic the effects of their parent cells but also possess many advantages such as high drug loading capacity, low immunogenicity, excellent biocompatibility, and low side effects. Currently, a total of 6 different dental stem cells (DSCs) including dental pulp stem cells (DPSCs), stem cells from exfoliated deciduous teeth (SHEDs), periodontal ligament stem cells (PDLSCs), dental follicle progenitor cells (DFPCs), stem cells from apical papilla (SCAPs) and gingival mesenchymal stem cells (GMSCs) have been isolated and identified. DSC-derived exosomes (DSC-Exos) are actively involved in intercellular communication, anti-inflammation, osteogenesis, angiogenesis, immunomodulation, nurturing neurons, and promoting tumor cell apoptosis. In this review, we will critically review the emerging role and clinical application potential of DSC-Exos.

## Introduction

Mesenchymal stem cells (MSCs) are multipotent progenitor cells that can be isolated from different tissues including but not limited to, bone marrow (Friedenstein et al., 1966), adipose tissue (Gruber et al., 2010), placenta (In 't Anker et al., 2004), umbilical cord (Secco et al., 2008), hair follicle (Bajpai, Mistriotis, and Andreadis 2012), palatine tonsil (Ryu et al., 2012), amniotic fluid (In 't Anker et al., 2003), fetal blood and liver (Campagnoli et al., 2001). Accumulative evidence has demonstrated that MSCs are capable of self-renewal, multipotent differentiation (Minguell et al., 2001; Munmun and Witt-Enderby 2021), regulating immune and inflammatory responses (Uccelli et al., 2008), and suppressing apoptosis and oxidative stress (Tsubokawa et al., 2010). More importantly, numerous pre-clinical studies have shown that MSCs hold promise in treating a wide range of diseases, including cancer, liver disease, cartilage repair, heart failure, stroke, neurological disorders, diabetes mellitus, autoimmune diseases, Duchenne muscular dystrophy, ocular surface diseases (Lodi et al., 2011; Lu et al., 2021). Over the past 10 years, more than 1000 MSC-based clinical trials have been conducted with recruitment of approximately 50,000 patients due to the safety of autogenous stem cells (Pittenger et al., 2019). Interestingly, accumulative evidence has suggested that the therapeutic effects of transplanted MSCs largely depend on the secretome of MSCs rather than the MSCs themselves (Phinney and Pittenger 2017).

Distinct from other types of cell therapy, MSC-based therapy achieves the therapeutic effects not only through direct cell-cell contacts but also by releasing secretome-derived bioactive factors (Levy et al., 2020). Recently, the MSC secreted extracellular vesicles (MSC-EVs), which include exosomes, microvesicles, and apoptotic bodies, have been suggested as a viable cell-free therapeutic alternative for MSCs (Jarrige et al., 2021). Compared to the cellular therapies, the MSC-EVs-based therapy offer many advantages such as high drug loading capacity, high specificity, low immunogenicity, excellent biocompatibility, high stability, lack of cytotoxicity, competitive price, and efficient intercellular communication. Therefore, MSC-EVs-based therapy, especially using exosomes, has emerged as a promising therapeutic tool for tissue repair and regeneration. In this review, we critically focus on the potential value of exosomes derived from dental stem cells (DSC-Exos) for treating oral and systemic diseases.

## Featured Properties of Dental Stem Cells and Exosomes

### Dental Stem Cells (DSCs)

**FIGURE 1:**
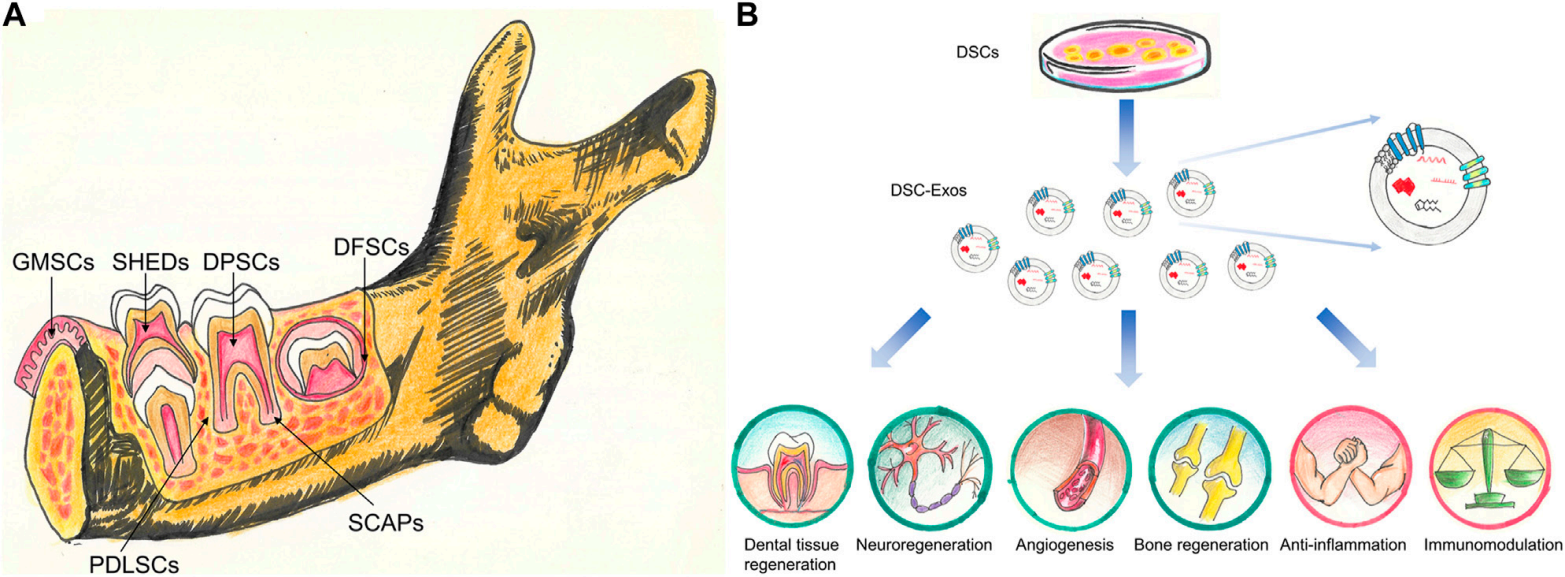
**(A)** Stem cell populations derived from different dental tissues. DPSCs, stem cells from dental pulp; SHEDs, stem cells from human exfoliated deciduous teeth; PDLSCs, stem cells from PDL; DFSCs, stem cells from dental follicle; SCAPs, stem cells from the apical papilla; GMSCs, stem cells from gingival tissues. **(B)** The potential clinical application of DSC-Exos. DSC-Exos hold great promise for dental tissue regeneration, neuroregeneration, angiogenesis, bone regeneration, anti-inflammation and immunomodulation.

Currently, a total of six different dental stem cells (DSCs) including dental pulp stem cells (DPSCs), stem cells from exfoliated deciduous teeth (SHEDs), periodontal ligament stem cells (PDLSCs), dental follicle progenitor cells (DFPCs), stem cells from apical papilla (SCAPs) and gingival mesenchymal stem cells (GMSCs) have been isolated and identified (Zhang et al., 2009; Sedgley and Botero 2012) (Figure 1A).

DPSCs, the first characterized DSCs, were isolated from the human dental pulp in 2000 (Gronthos et al., 2000). The MSC-like properties of DPSCs enable them to differentiate into multiple cell lineages such as neural-like cells, osteoblasts, adipocytes, and chondrocytes, and form mineralized tissue, blood vessels, and nerve tissues *in vivo* (Gronthos et al., 2000; Gronthos et al., 2002; Victor and Reiter 2017). Besides, DPSCs have also been demonstrated to regenerate dentin and functional dental pulp with vasculature and nerves (Iohara et al., 2014).

SHEDs, obtained from human exfoliated deciduous teeth by Miura et al. (2003), is a population of highly proliferative cells capable of differentiating into odontoblasts and endothelial cells (Miura et al., 2003). After being injected into human root canals, the transplanted SHEDs could differentiate into functional odontoblasts and form dentin-like tissues (Rosa et al., 2013). Interestingly, SHEDs remained viable following transplantation into the mouse brain and expressed neural markers (Miura et al., 2003).

Similarly, PDLSCs are named according to their tissue of origin, and isolated from the periodontal ligament which is a soft connective tissue between the teeth and alveolar bone. PDLSCs are promising seed cells for the restoration of periodontal tissues and are capable of differentiating into adipocytes, chondrocytes, osteoblasts, cardiac myocytes and neural cells (Gay et al., 2007; Tomokiyo et al., 2019). PDLSCs interact tightly with the periodontitis niche in a positive feedback loop. The injured PDLSCs may aggravate the disrupted periodontal tissue homeostasis, while bacterial infections and subsequent host immune responses affect the functional properties of resident PDLSCs by shaping the periodontal microenvironment (Zhang Y. et al., 2021).

DFPCs were first isolated from the dental follicle surrounding the impacted third molar tooth germ and identified by Morsczeck et al. (2005). DFPCs not only have better immunomodulatory and anti-apoptotic effects on the immune system than DPSCs and SHED, but also exhibit greater osteogenic properties than SHED and DPSCs as the osteogenic-related markers such as Runx2 and DSPP are highly expressed in DFPCs (Zhu et al., 2018). In addition, DFPCs were able to differentiate into cementoblasts *in vivo* (Handa et al., 2002). Therefore, DFSCs are a promising alternative source for dental hard tissue regeneration.

SCAPs are isolated from the apical papilla of immature permanent teeth, and play a critical role in tooth root development and dentin regeneration (Sonoyama et al., 2008). The high telomerase activity of SCAPs makes them a better choice of dentin regeneration compared to DPSCs (Sonoyama et al., 2006). In addition, SCAPs are able to form cementum/PDL-like complex *in vivo* (Han et al., 2010). Moreover, SCAPs have been shown to be low immunogenicity and possess immunomodulatory functions, which make them an attractive and promising therapeutic tool for tissue regeneration (Ding et al., 2010).

GMSCs were first isolated from healthy gingival tissues and characterized by Zhang et al., in 2009 (Zhang et al., 2009). GMSCs can differentiate into adipocytes, chondrocytes, and endothelial cells, and have shown great promise for nerve regeneration. Besides, GMSCs exerted anti-proliferative and pro-apoptotic effects on oral cancer cells both *in vitro* and *in vivo* (Ji et al., 2016).

To the best of our knowledge, it is still extremely difficult to distinguish various sources of DSCs on a molecular level. All different types of DSCs are derived from migrating neural crest cells, which are originated from the embryonic ectoderm germ layer (Pisciotta et al., 2020). The fibroblast-like DSCs share similar surface marker expression profiles, and have high clonogenic potential and multipotent differentiation capacity. A recent study suggested that calreticulin might be a promising biomarker for distinguishing DPSCs and GMSCs. We have summarized the similarity, advantages, and weaknesses of each type of DSCs in Table 1.

**TABLE 1:** The common and different properties for each type of DSCs.

Cell type	Advantage	Weakness	Similarity
DPSCs	Formation of dentin–pulp-like complex, Source for reparative dentin	Essential to be extracted from healthy adult teeth, differential growth rates, cell morphologies, and sizes	Fibroblast-like morphology
SHEDs	Formation of dentin-like tissue or pulp-like tissue, The most proliferative DSCs	Unable to form dentin-pulp-like complex	Common markers: CD13^+^, CD29^+^, CD73^+^, CD90^+^, CD105+, CD106+, CD146+, Stro-1+, CD34^−^, CD45^−^, CD11b-, CD14^−^, CD19^−^, CD79a-, and HLA-DR-High clonogenic potential
PDLSCs	Formation of PDL-cementum-like construction	Lack odontogenic potential	Multilineage differentiation capacity
DFPCs	Formation of alveolar bone, Formation of PDL-cementum-like construction	Lack odontogenic potential	
SCAPs	Maintenance of root maturation, Formation of dentin-pulp-like complex	Not easily obtainable	
GMSCs	Easy to isolate, Long-term stability	Lack odontogenic potential	

### Exosomes

Exosomes, with a size range of 40–160 nm (average 100 nm), are originated from the endosomal system by inward budding of the endosomal membrane (Xu F. et al., 2020; Yoon et al., 2020). Ultracentrifugation, size-based isolation techniques, immunoaffinity capture-based techniques, exosome precipitation, and microfluidic-based isolation techniques are utilized to isolate exosomes (Doyle and Wang 2019). They are found in abundance in body fluids including breast milk, saliva, blood, and urine (Vlassov et al., 2012). This type of extracellular vesicles was initially thought of as waste products (Wolf 1967). However, accumulative evidence has demonstrated that exosomes carry many bioactive molecules including nucleic acids, proteins, lipids, metabolites (Zhang Y. et al., 2020; Kalluri and LeBleu 2020). These encapsulated materials are transported to neighboring or distant cells selectively, which contribute to cell-cell communication, signal transduction, immune response modulation, antigen presentation, and epigenetic reprogramming of recipient cells (Janockova et al., 2021). The biological functions of exosomes are heavily dependent on physiological/pathological conditions of originating tissues or cells at the time of exosome secretion, and the surface receptors of the recipient cells (Alcayaga-Miranda et al., 2016). As shown in Figure 1B, the DSC-derived exosomes might represent an ideal therapeutic tool for tissue repair and regeneration as well as treating other systemic diseases.

## Translational and Clinical Applications of Dental Stem Cell-Derived Exosomes

### DPSC-Exos

The high osteo/odonto-induction capability and easy availability of DPSCs-derived exosomes (DPSC-Exos) make them highly attractive in regenerative medicine (Imanishi et al., 2021). In addition, aged DPSCs still have active cellular metabolism and secrete functional exosomes which can penetrate the blood-brain barrier, indicating DPSC-Exos might be an effective drug carrier for the treatment of various diseases, especially for neurological disorders like Parkinson’s disease (PD) (Haney et al., 2015; Iezzi et al., 2019). For instance, DPSC-Exos were deemed as a suitable carrier to deliver tumor suppressor miR-34a to inhibit the proliferation of breast cancer cells (Vakhshiteh et al., 2021). In terms of odontogenic differentiation, Huang et al. revealed that DPSC-Exos attached to biomaterials by binding to matrix proteins like fibronectin and type I collagen (Huang et al., 2016). Besides, DPSC-Exos, especially those isolated from DPSCs cultured under an odontogenic differentiation environment, increased the expression of genes indispensable for odontogenic differentiation of naïve DPSCs *in vitro* and promoted the regeneration of dental pulp-like tissues *in vivo* (Huang et al., 2016; Swanson et al., 2020). Similarly, compared to exosomes isolated from DPSCs cultured undergrowth state, DPSC-Exos obtained under odontogenic conditions exhibited better performance for trigging odontogenic differentiation of DPSCs by activating the TGF-β1/smads signaling pathway (Huang et al., 2016; Hu et al., 2019). Exosomes derived from both mineralizing DPSCs and an immortalized murine odontoblast cell line (MDPC-23) were superior to traditional glass-ionomer cement for forming the reparative dentin bridge (Swanson et al., 2020). It seems that lipopolysaccharide (LPS) treatment can significantly alter the biological functions of DPSCs-Exos. Li et al. demonstrated that LPS-preconditioned DPSC derived exosomes (LPS-DPSC-Exos) promoted the proliferation, migration, and odontogenic differentiation of Schwann cells (Li et al., 2021). Similarly, LPS-DPSC-Exos were shown to promote angiogenesis by facilitating the proliferation, migration, and tube formation abilities of human umbilical vein endothelial cells (HUVECs) *in vitro* through changing the microRNA (miRNA) expression profile and increasing the levels of kinase-insert domain-containing receptor and vascular endothelial growth factor (Huang et al., 2021). DPSC-Exos also exhibits strong anti-inflammatory and immunomodulatory effects. For instance, DPSC-Exos facilitated alveolar bone reconstruction and periodontal epithelium healing in a mouse model of periodontitis *via* delivering exosomal miR-1246 (Shen et al., 2020). DPSC-Exos exerted immunomodulatory effects by suppressing the differentiation of CD4+T cells into T helper 17 cells (Th17) and facilitated the transformation of CD4+T cells into regulatory T cells (Tregs), leading to the increased level of anti-inflammatory cytokines (Ji et al., 2019). In addition to oral diseases, DPSC-Exos based therapy is promising for treating systemic diseases. For example, exosomes derived from DPSC might be more suitable in the treatment of neurodegenerative diseases than MSCs from mesodermal tissues such as bone marrow or adipose tissues (Wang et al., 2019). Exosomes secreted from miR-140-5p overexpressing DPSCs promoted the expression of genes related to chondrogenic differentiation and exerted anti-apoptotic effects both *in vitro* and *in vivo*, which represented a potentially novel therapeutic strategy for osteoarthritis (Lin et al., 2021).

### SHED-Exos

The applications of SHED-derived exosomes (SHED-Exos) can be classified into the following three categories: promotion of osteogenesis, neurotrophic property and anti-inflammatory function. In terms of osteogenesis, SHED-Exos promoted osteogenic differentiation of PDLSCs by upregulating key genes and signaling pathways related to osteogenesis (Wang A. et al., 2020). Similarly, SHED-Exos was shown to have the potency for mobilizing naïve BMMSCs, resulting in enhancing bone regeneration (Luo et al., 2021). Wei et al. revealed that SHED-Exo promoted osteogenesis and suppressed adipogenesis of bone marrow mesenchymal stem cells (BMMSCs) by decreasing lipid droplets and the expression of the adipogenic marker PPARγ (Wei et al., 2020). SHED-Exos promoted neovascularization of HUVECs and osteogenic differentiation of BMMSCs, and this regulatory effect could be counteracted by adding AMPK inhibitor, indicating that the AMPK signaling pathway might involve in mediating the pro-angiogenic effects and pro-bone regeneration activities of SHED-Exos (Wu et al., 2019). Concerning neurotrophic property, exosomes isolated from SHEDs grown on the laminin-coated three-dimensional alginate micro-carriers protected dopaminergic neurons from 6-hydroxy-dopamine induced apoptosis, whereas exosomes from SHEDs grown under standard culture conditions had no such effects (Jarmalaviciute et al., 2015). Li et al. injected SHED-Exos into the traumatic brain injury (TBI) rat model and observed that SHED-Exos contributed to rat motor functional restoration and cortical lesion reduction by shifting microglia polarization (Li et al., 2017). Narbute et al. showed that SHED-Exos significantly improved the gait impairments and contralateral rotations in the unilateral 6-hydroxydopamine (6-OHDA) rat model of PD (Narbute et al., 2019). Collectively, SHED-Exos are promising therapeutic tools for neurological disorders like PD. In terms of anti-inflammatory effect, SHED-Exos significantly suppressed the carrageenan-induced acute inflammation *in vivo* (Pivoraite et al., 2015). Similarly, Luo et al. showed that SHED-Exos markedly reduced the inflammation in chondrocytes derived from the temporomandibular joint through delivering miR-100-5p (Luo et al., 2019).

### PDLSC-Exos

The major regulatory functions of PDLSCs-derived exosomes (PDLSC-Exos) include angiogenesis, anti-inflammation, and osteogenesis. Inflammation led to increasing exosome secretion in PDLSCs, and exosomes derived from inflamed PDLSC promoted angiogenesis of HUVECs by upregulating the expression of vascular specific marker CD31 and VEGFA (Zhang Z. et al., 2020). The exosomes isolated from PDLSCs that were exposed to the LPS-induced periodontitis environment demonstrated good anti-inflammatory ability by modulating the balance of T helper cell 17 (Th17)/regulatory T cell (Treg) through the miR-155-5p/SIRT1 pathway (Zheng et al., 2019). In terms of osteogenesis, PDLSC-Exos possess the capacity for inducing osteogenic differentiation of BMMSCs *via* regulating AMPK signaling, MAPK signaling, and insulin signaling pathways (Liu et al., 2020). P2X7R overexpressing PDLSCs-derived conditional medium and exosomes markedly improved the osteogenic capacity of PDLSCs in the inflammatory microenvironment by delivering miR-3679-5p, miR-6515-5p, and miR-6747-5p (Xu X. Y. et al., 2020). Interestingly, the circRNA and lncRNA expression profile was significantly altered in PDLSCs-exos during the osteogenic differentiation of PDLSCs, indicating that the exosomal non-coding RNAs might play a critical role in regulating PDLSCs osteogenesis (Xie et al., 2021). Collectively, PDLSC-Exos is beneficial to the maintenance of periodontal homeostasis by promoting proliferation, angiogenesis, and osteogenesis as well as regulating the inflammatory responses.

### SCAP-Exos

SCAP-derived exosomes (SCAP-Exos) also show great potential for oral tissue regeneration. Zhuang et al. demonstrated that SCAP-Exos promoted the dentinogenesis of BMMSCs both *in vitro* and *in vivo*, indicating that SCAP-Exos might represent a potential therapeutic tool for dentine-pulp complex regeneration (Zhuang et al., 2020). Through injecting into the palatal gingival complex critical-size defects (CSD) of mice, SCAP-Exos significantly improved angiogenesis and soft tissue regeneration (Liu et al., 2021). In terms of mechanism, SCAP-Exos promoted filopodium formation, migration, and cytoskeletal reorganization of endothelial cells *via* delivering exosomal Cdc42. Wang et al. compared the piRNA expression profiles between SCAP-Exos and BMMSC-Exos (Wang M. et al., 2020). The differentially expressed piRNAs were found to be closely associated with many important biological functions such as catalytic activity, metabolic processes, cellular processes, binding, and biological regulation, suggesting that piRNAs might play a crucial role in regulating the molecular activities of exosomes.

### GMSC-Exos

Although currently few studies are available regarding the potential therapeutic applications of GMSC-Exos, they hold a great promise for tissue regeneration. The pre-osteoblasts MC3T3-E1 treated with GMSC-Exos were found deep Alizarin red staining, increased ALP activity, and upregulated expression of osteogenic genes, suggesting that GMSC-Exos facilitated the osteogenic differentiation of MC3T3-E1 (Jiang and Xu 2020). The exosomes isolated from tumor necrosis factor-alpha (TNF-α) preconditioned GMSC-Exos induced the polarization of anti-inflammatory M2 macrophage by improving the secretion of GMSC-Exos and increased the exosomal expression of CD73 (Nakao et al., 2021). In a high-lipid microenvironment, GMSC-Exos suppressed lipid accumulation, transformed pro-inflammatory macrophages into anti-inflammatory phenotype, and decrease the secretion and expression of inflammatory factors including IL-6, IL-1β, TNF-α, and cluster of differentiation 86 (Zhang Z. et al., 2021). Besides, GMSC-Exos presented anti-osteoclastogenic activity and suppressed inflammatory bone loss by delivering miR-1260b (Nakao et al., 2021). Rao et al. combined GMSC-Exos with biodegradable chitin conduits and injected the composite into the rat sciatic nerve defect model. The results showed that GMSC-Exos enhanced the proliferation of Schwann cells and the growth of the dorsal root ganglion neuron axon as well as promoting the formation of nerve fibers and myelin, which subsequently contributed to restoring motor skills, nerve conduction function, and muscle movement (Rao et al., 2019). GMSC-Exos also promoted healing of diabetic skin defects by facilitating re-epithelialization, collagen remodeling, angiogenesis, and nerve growth in a diabetic rat skin defect model (Shi et al., 2017). Combining GMSC-Exos with small intestinal submucosa-extracellular matrix promoted taste bud regeneration and tongue lingual papillae recovery in a rat tongue defect model (Zhang et al., 2019). We have summarized the currently available evidence regarding the potential clinical application of DSC-Exos (Table 2).

**TABLE 2:** The potential clinical application of DSC-Exos.

Origin	Administration	Recipients	Application	Refs	The potentially most suitable application of specific DSC-Exos
DPSCs	*In vitro*	Breast cancer cells	Cancer	Vakhshiteh et al. (2021)	Pulp and dentin regeneration
—	*In vitro*	CD4^+^ T cells	Immunomodulation	Ji et al. (2019)	
—	*In vitro*	Schwann cells	Pulp regeneration	Li et al. (2021)	
—	*In vitro*	HUVECs	Angiogenesis	Huang et al. (2021)	
—	*In vivo*	Athymic nude mice	Pulp regeneration	Huang et al. (2016)	
—	*In vivo*	Rat pulpotomy model	Dentin regeneration	Swanson et al. (2020)	
—	*In vivo*	Periodontitis model	Periodontitis	Shen et al. (2020)	
—	*In vivo*	Osteoarthritis model	Osteoarthritis	Lin et al. (2021)	
SHEDs	*In vitro*	PDLSCs	Osteogenic differentiation	Wang et al. (2020b)	Parkinson’s disease
—	*In vitro*	BMMSCs	Migration promotion	Luo et al. (2021)	Neuroregeneration
—	*In vitro*	Neurons	Parkinson’s disease	Jarmalaviciute et al. (2015)	
—	*In vitro*	Chondrocytes	Osteoarthritis	Luo et al. (2019)	
—	*In vivo*	Periodontal defect	Bone regeneration	Wu et al. (2019)	
—	*In vivo*	BMMSCS	Bone regeneration	Wei et al. (2020)	
—	*In vivo*	Traumatic brain injury model	Traumatic brain injury	Li et al. (2017)	
—	*In vivo*	Parkinson’s disease model	Parkinson’s disease	Narbute et al. (2019)	
—	*In vivo*	Mouse paw edema	Anti-inflammation	Pivoraite et al. (2015)	
PDLSCs	*In vitro*	CD4^+^ T cells	Immunomodulation	Zheng et al. (2019)	Periodontitis induced bone loss
—	*In vitro*	BMMSCs	Osteogenic differentiation	Liu et al. (2020)	
—	*In vitro*	PDLSCs	Osteogenic differentiation	Xu et al. (2020b)	
—	*In vitro*	HUVECs	Angiogenesis	Zhang et al. (2020a)	
SCAPs	*In vivo*	Immunodeficient mice	Dentinogenesis	Zhuang et al. (2020)	Not enough evidences for evaluation
—	*In vivo*	Critical-size defects	Soft tissue regeneration	Liu et al. (2021)	
GMSCs	*In vitro*	Pre-osteoblast	Osteogenic differentiation	Jiang and Xu (2020)	Taste bud regeneration and would healing
—	*In vitro*	Macrophages	Anti-inflammation	Zhang et al. (2021b)	
—	*In vivo*	Schwann and DRG cells	Nerve repair	Rao et al. (2019)	
—	*In vivo*	Skin defect model	Would healing	Shi et al. (2017)	
—	*In vivo*	Periodontitis model	Bone regeneration	Nakao et al. (2021)	
—	*In vivo*	Tongue defect model	Taste bud regeneration	Zhang et al. (2019)	
DFPCs	NA	NA	Not evaluated yet	NA	NA

## Future Perspectives and Challenges

DSC-Exos hold great promise in tissue repair and regeneration as well as treating other diseases. However, to the best of our knowledge, there is still not enough evidence for evaluating and comparing the molecular differences, biological functions, and therapeutic applications among different types of DSC-Exos as well as between DSC-Exos and other MSCs-Exos. Compared to exosomes derived from other MSCs, DSC-Exos might possess their own advantages. For instance, DPSC-Exos have stronger immunomodulatory, anti-necrotic, and anti-apoptotic effects than BMMSC-Exos (Venugopal et al., 2018; Ji et al., 2019). The currently available evidence demonstrates that DSC-Exos might share similar limitations and weaknesses. Compared to the exosomes from BMMSCs and adipose tissue-derived MSCs, the isolation of a sufficient amount of DSCs-Exos is still an obstacle for hindering their therapeutic applications (Stanko et al., 2018). More importantly, compared to autologous BMMSC from bone marrow aspirate, DSCs are mainly collected from non-renewable exfoliated deciduous teeth, third molars, and teeth extracted by orthodontic treatment and irreversible periodontitis, or discarded tissues after dental surgery. Therefore, it is challenging to obtain DSC-Exos in time when they are needed. Only under the condition that the unnecessary tissues like exfoliated deciduous teeth and third molar were stored previously by a stable and long-term approach, the applications of DSC-Exos might be popularized on a large scale. In addition, there are a lack of standardized and accepted approaches for storage, transport and large-scale production of DSCs-Exos, which has significantly affected their clinical applications. Mover, determining the most appropriate dose of exosomes under different pathological conditions, avoiding the off-target effects, and ensuring sufficient biological safety of DSC-Exos need to be urgently addressed in the coming pre-clinical studies and/or clinical trials.

## Conclusions

DSCs mainly exert their therapeutic effects by the secretion of exosomes *via* a paracrine mechanism. Compared to DSCs, DSC-Exos possess unique advantages such as high drug loading capacity, high specificity, low immunogenicity, excellent biocompatibility, easily obtainable, low side effects, and nanoscale size. In addition, DSC-Exos have been shown to regulate many important biological processes including intercellular communication, anti-inflammation, osteogenesis, angiogenesis, immunomodulation, nurturing neurons, and promoting tumor cell apoptosis. Although there are still many barriers to translation into clinical practice, DSC-Exos is emerging as a promising and practical therapeutic approach for tissue repair and regeneration.
